# Supplementary material for: Structural and functional characterization of TgGSK3, a druggable kinase in Toxoplasma gondii
Source: Nat Commun. 2025 Nov 5;16:9765. doi: 10.1038/s41467-025-64701-7 (PMC12589562; doi:10.1038/s41467-025-64701-7)
Supplement: Supplementary file 1 — Supplementary Information [file 41467_2025_64701_MOESM1_ESM.pdf]

Supplementary Table 1. Data collection and refinement statistics.

	Monomeric TgGSK3/LY2090314 (aa 46-385)	Tetramer of TgGSK3/LY2090314 (aa 21-385)
**PDB id**	9HVX	9HW6
**Wavelength**	0.9655	0.9655
**Resolution range**	46.16 - 2.1 (2.38 - 2.1)	45.48 - 2.9 (3.004 - 2.9)
**Space group**	P 21 21 2	P 1 21 1
**Unit cell**	56 120.84 49.95 90 90 90	70.91 161.73 83.28 90 92.21 90
**Total reflections**	121322 (31146)	135277 (13334)
**Unique reflections**	20469 (3232)	40947 (4080)
**Multiplicity**	5.93 (5.42)	3.3 (3.4)
**Completeness (%)**	99.81 (98.62)	98.50 (98.84)
**Mean I/sigma(I)**	8.4 (1.4)	4.86 (1.5)
**Wilson B-factor**	34.89	60.85
**CC1/2**	99.6 (55.6)	96.2 (55)
**Reflections used in refinement**	20463 (1997)	40911 (4077)
**Reflections used for R-free**	1999 (195)	1996 (204)
**R-work**	0.1981 (0.3403)	0.2036 (0.2963)
**R-free**	0.2505 (0.3809)	0.2598 (0.3335)
**Number of non-hydrogen atoms**	2748	10772
**macromolecules**	2589	10610
**ligands**	41	162
**solvent**	118	0
**Protein residues**	323	1320
**RMS(bonds)**	0.009	0.011
**RMS(angles)**	1.27	1.28
**Ramachandran favored (%)**	97.12	91.68
**Ramachandran allowed (%)**	2.88	6.38
**Ramachandran outliers (%)**	0.00	1.94
**Rotamer outliers (%)**	1.74	6.47
**Clashscore**	4.00	10.58
**Average B-factor**	33.01	60.47
**macromolecules**	33.09	60.71
**ligands**	24.32	44.99
**solvent**	34.25	-

Statistics for the highest-resolution shell are shown in parentheses.

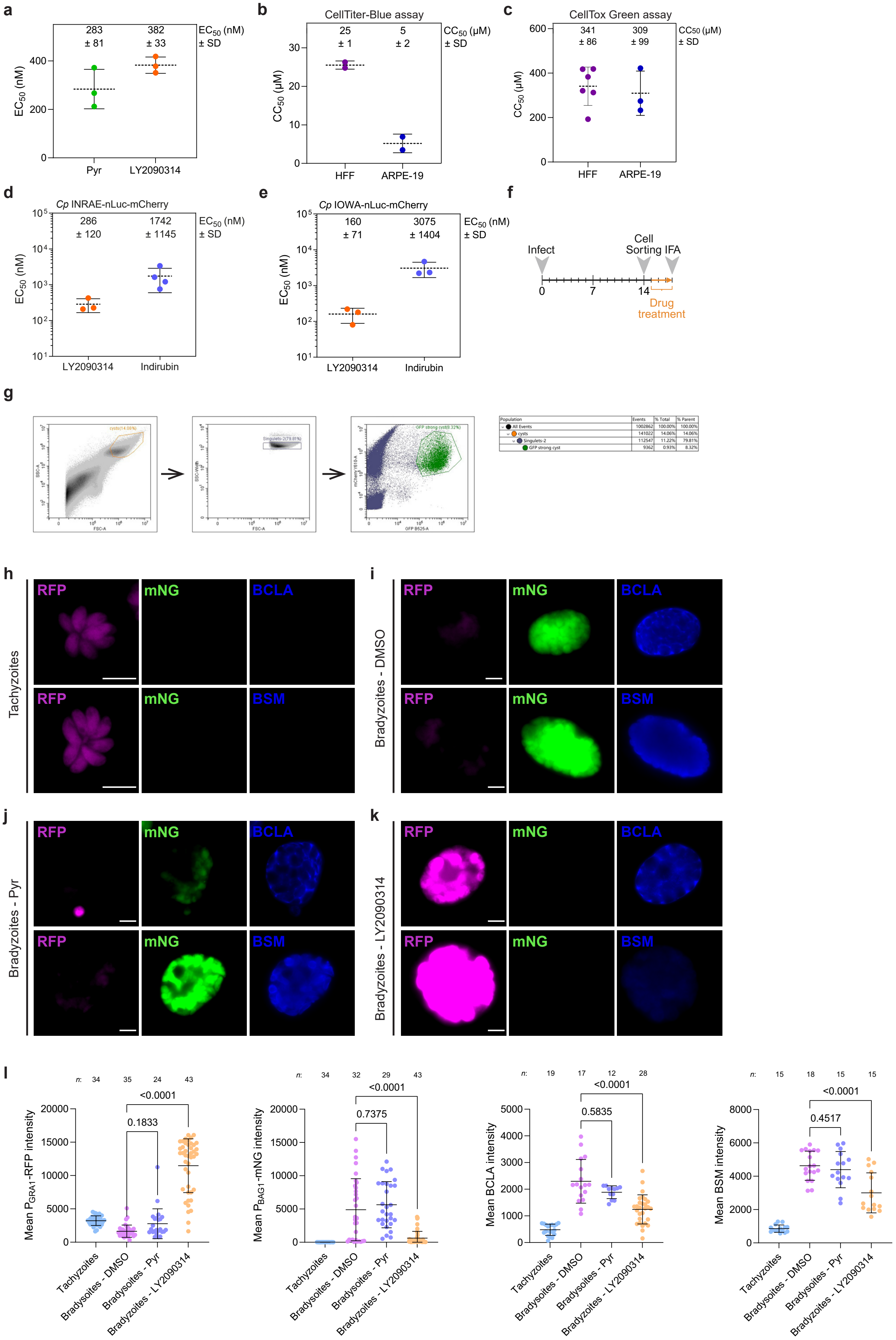
a
283 ± 81
382 ± 33
EC50 (nM) ± SD
EC50 (nM)
Pyr
LY2090314
b
CellTiter-Blue assay
25 ± 1
5 ± 2
CC50 (µM) ± SD
CC50 (µM)
HFF
ARPE-19
c
CellTox Green assay
341 ± 86
309 ± 99
CC50 (µM) ± SD
CC50 (µM)
HFF
ARPE-19
d
Cp INRAE-nLuc-mCherry
286 ± 120
1742 ± 1145
EC50 (nM) ± SD
EC50 (nM)
LY2090314
Indirubin
e
Cp IOWA-nLuc-mCherry
160 ± 71
3075 ± 1404
EC50 (nM) ± SD
EC50 (nM)
LY2090314
Indirubin
f
Infect
Cell Sorting
IFA
0
7
14
Drug treatment
g
h
Tachyzoites
RFP
mNG
BCLA
BSM
i
Bradyzoites - DMSO
j
Bradyzoites - Pyr
k
Bradyzoites - LY2090314
l
Mean PGRA1-RFP intensity
Mean PBAG1-mNG intensity
Mean BCLA intensity
Mean BSM intensity
n:
34 35 24 43
34 32 29 43
19 17 12 28
15 18 15 15
<0.0001
0.1833
0.7375
0.5835
0.4517
Tachyzoites
Bradysoites - DMSO
Bradysoites - Pyr
Bradyzoites - LY2090314

Supplementary Figure 1. Effect of LY2090314 treatment on *T. gondii* bradyzoites. (a) The half-maximal effective concentration (EC_{50}) values for pyrimethamine and LY2090314 against *T. gondii* parasites. A confluent human foreskin fibroblasts (HFFs) monolayer was infected with tachyzoites of the *T. gondii* RH NLuc strain (Supplementary Data 1). The EC_{50} values of each biological replicate were determined by non-linear regression analysis. EC_{50} data are presented as means ± SD from n = 3 independent biological replicates, each with three technical replicates. (b-c) The half-maximal cell cytotoxicity concentration (CC_{50}) values for LY2090314 were determined using the CellTiter-Blue (b) or CellTox Green (c) assay kits on HFF and ARPE-19 human cells. The CC_{50} values of each biological replicate were determined by non-linear regression analysis. CC_{50} data are presented as means ± SD from n = 2 in (b), and n = 6 and n = 3 in (c) independent biological replicates for HFF cells and ARPE-19, respectively, each performed with three technical replicates. (d-e) EC_{50} values for LY2090314 and indirubin against *C. parvum* parasites. Mean EC_{50} values ± SD from at least three independent biological replicates are shown. (f) Schematic timeline of tachyzoite-to-bradyzoite differentiation and drug treatment. The ME49 pGRA1-dsRed2.0 pBAG1-mNeonGreen reporter strain was cultured under bradyzoite-inducing conditions. After 14 days, cysts were isolated by fluorescence-activated cell sorting, and the following day, sorted cysts were treated with either DMSO (vehicle control), 2 µM pyrimethamine, or 600 nM LY2090314. Following 72 hours of treatment, cells were fixed and processed for immunofluorescence microscopy. (g) Gating strategy for FACS sorting of cysts. (h-k) Representative fluorescence microscopy images of the bradyzoite reporter strain (ME49 pGRA1-dsRed2.0 pBAG1-mNeonGreen) maintained under tachyzoite growth conditions (h) or bradyzoite-inducing conditions treated with DMSO (i), 2 µM pyrimethamine (j), or 600 nM LY2090314 (k) for 72 hours. Cells were fixed and stained with antibodies against the bradyzoite markers BCLA and BSM (blue). RFP and mNG correspond to P_{GRA1}-driven dsRed2.0 (magenta) and P_{BAG1}-driven mNeonGreen (green), respectively. Scale bars, 5 µm. (l) Quantification of fluorescence intensities for RFP, mNeonGreen, BCLA, and BSM signals from the images shown in panels (h–k). Data are mean value ± SD from n = 6 (GRA1 and BAG1) and n = 3 (BCLA and BSM) biological replicates. The number of vacuoles/cysts analyzed is indicated above the figures. *P*-values were calculated using two-way ANOVA with Dunnett's multiple-comparison *post hoc* test with the DMSO-treated bradyzoites.

a

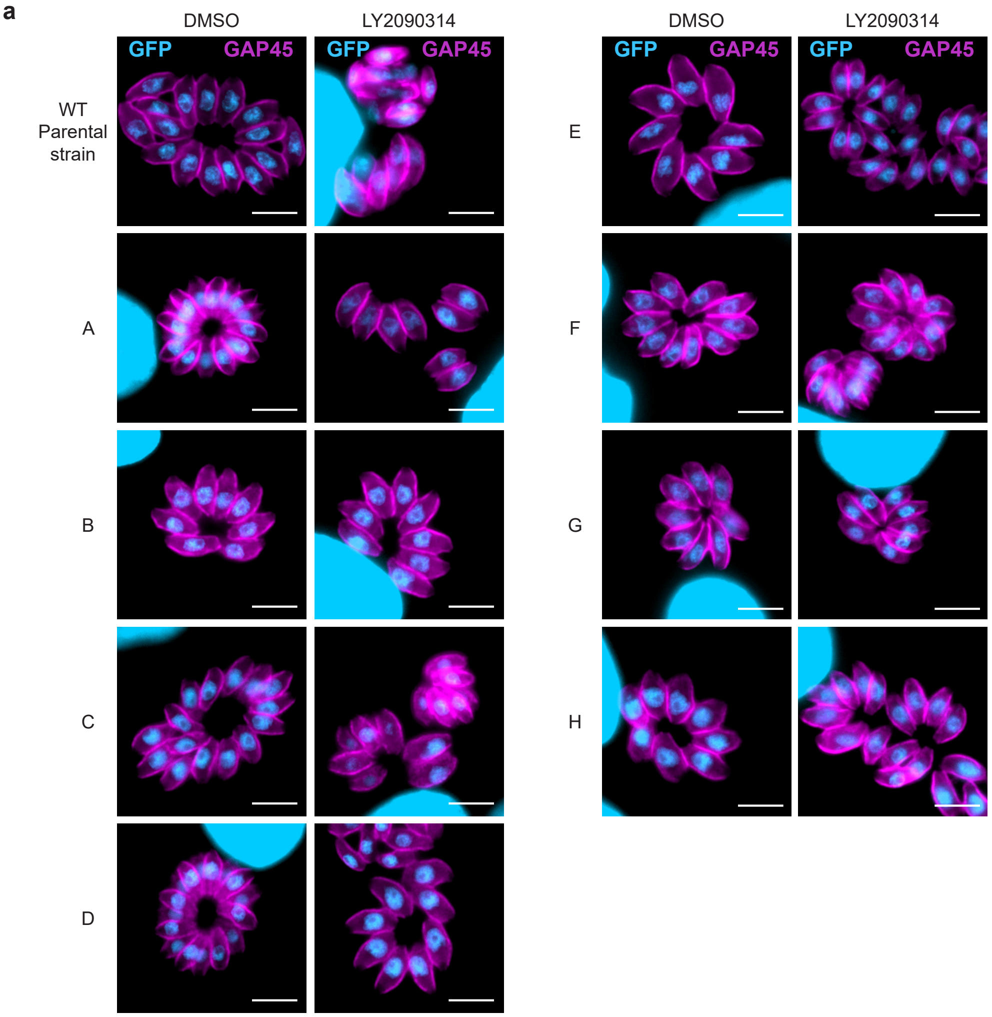

b

```
                                       20                                        40                                        60                                        80
TgGSK3    ----------  ---------M  ----------  ----------  -------PDP  -------QYD  PAAAQ-----  ----------  12
CpGSK3    ----------  ----------  ----------  ----------  ----------  -------MSN  VNVQLSRKQQ  LLERGCNEMI  23
PfGSK3    -MKNWPIDED  INIYEEKNHT  ----------  ----------  -------NNK  -------NYV  NNFEMS----  ---------D  32
HsGSK3α   MSGGGPSGGG  PGGSGRARTS  SFAEPGGGGG  GGGGGPGGSA  SGPGGTGGGK  ASVGAMGGGV  GASSSGGGPG  GSGGGGSGGP  80
HsGSK3β   ----------  ----------  ----------  ----------  ----------  ----------  ---MSGRPRT  TSFAESCKPV  17

                                      100                                       120                                       140                                       160
TgGSK3    KSSNARGGGS  QRTTAVKGER  -Q---SRVHT  GNPHPTLTYD  IGRVLGNGSF  GVVTEARCVD  TGEVVAIKKV  LQDPRYKNRE  88
CpGSK3    EEKTNLTNLN  HNVTLSSGLS  TT---ATSST  NLETSSKKYS  LGKTLGTGSF  GIVCEVFDIE  SGKRFALKKV  LQDPRYKNRE  100
PfGSK3    QKDEEEYSHS  SNRSEDEDEE  -R---TIDNE  INRSPNKSYK  LGNIIGNGSF  GVVYEAICID  TSEQVAIKKV  LQDPQYKNRE  108
HsGSK3α   GAGTSFPPPG  VKLGRDSGKV  TTVVATLGQG  PERSQEVAYT  DIKVIGNGSF  GVVYQARLAE  TRELVAIKKV  LQDKRFKNRE  160
HsGSK3β   QQPSAFGSMK  VSRDKDGSKV  TTVVATPGQG  PDRPQEVSYT  DTKVIGNGSF  GVVYQAKLCD  SGELVAIKKV  LQDKRFKNRE  97

                                      180                                       200                                       220                                       240
TgGSK3    LDIMKELKHP  NVVKLIDYFY  TEMGERDS--  ----------  ----------  ------NDHN  RFLNVVMEHI  PETVYRVMKS  140
CpGSK3    LDIMKVLDHV  NIIKLVDYFY  TTGDEEPKPP  QPPDDHNKLG  GKNNGVNNHH  KSVIVNPSQN  KYLNVIMEYV  PDTLHKVLKS  180
PfGSK3    LMIMKNLNHI  NIIYLKDYYY  TESFKK----  ----------  ----------  ------NEKN  IFLNVVMEYI  PQTVHKYMKY  158
HsGSK3α   LQIMRKLDHC  NIVRLRYFFY  SSGEKK----  ----------  ----------  --------DE  LYLNLVLEYV  PETVYRVARH  208
HsGSK3β   LQIMRKLDHC  NIVRLRYFFY  SSGEKK----  ----------  ----------  --------DE  VYLNLVLDYV  PETVYRVARH  145

                                      260                                       280                                       300                                       320
TgGSK3    FLRANQQVPF  ILIKLYTYQM  CRALGYLHAL  GICHRDIKPQ  NLLVDSRTHV  LKLCDFGSAK  RLVPGEQSVS  YICSRFYRAP  220
CpGSK3    FIRSGRSIPM  NLISIYIYQL  FRAVGFIHSL  GICHRDIKPQ  NLLVNSKDNT  LKLCDFGSAK  KLIPSEPSVA  YICSRFYRAP  260
PfGSK3    YSRNNQALPM  FLVKLYSYQL  CRALSYIHSK  FICHRDLKPQ  NLLIDPRTHT  LKLCDFGSAK  NLLAGQRSVS  YICSRFYRAP  238
HsGSK3α   FTKAKLTIPI  LYVKVYMYQL  FRSLAYIHSQ  GVCHRDIKPQ  NLLVDPDTAV  LKLCDFGSAK  QLVRGEPNVS  YICSRYYRAP  288
HsGSK3β   YSRAKQTLPV  IYVKLYMYQL  FRSLAYIHSF  GICHRDIKPQ  NLLLDPDTAV  LKLCDFGSAK  QLVRGEPNVS  YICSRYYRAP  225

                                      340                                       360                                       380                                       400
TgGSK3    ELMLGASEYT  TAIDVWSIGC  VLGELLLGRP  LFAGETSVDQ  LVKIIQILGT  PSRRQMSTMN  PNYTEFRFPD  VKPREWKSIF  300
CpGSK3    ELMLGATEYT  PSIDLWSIGC  VFGELILGKP  LFSGETSIDQ  LVRIIQIMGT  PTKEQMIRMN  PHYTEVRFPT  LKAKDWRKIL  340
PfGSK3    ELMLGSTNYT  THIDLWSLGC  IIAEMILGYP  IFSGQSSVDQ  LVRIIQVLGT  PTEDQLKEMN  PNYADIKFPD  VKSKDLRKVF  318
HsGSK3α   ELIFGATDYT  SSIDVWSAGC  VLAELLLGQP  IFPGDSGVDQ  LVEIIKVLGT  PTREQIREMN  PNYTEFKFPQ  IKAHPWTKVF  368
HsGSK3β   ELIFGATDYT  SSIDVWSAGC  VLAELLLGQP  IFPGDSGVDQ  LVEIIKVLGT  PTREQIREMN  PNYTEFKFPQ  IKAHPWTKVF  305

                                      420                                       440                                       460                                       480
TgGSK3    ASHIASTETN  DPSWDQALDL  LTKFLRYEPG  ERLLPLEALA  HDFFDELRLP  ST-------R  LPSG--GPLP  ELFTFSQLEL  371
CpGSK3    PEGT------  ---PSLAIDL  LEQILRYEPD  LRINPYEAMA  HPFFDHLRNS  YESEVKNNSN  FPHGVNQNIP  QLFNFSPYEL  411
PfGSK3    PKGT------  ---PDEAINL  ITQFLKYEPL  KRLNPIEALA  DPFFDELRDP  CI-------K  LPKYI-DKLP  ELFNFCKEEI  381
HsGSK3α   KSRT------  ---PPEAIAL  CSSLLEYTPS  SRLSPLEACA  HSFFDELRCL  GT-------Q  LPNN--RPLP  PLFNFSAGEL  430
HsGSK3β   RPRT------  ---PPEAIAL  CSRLLEYTPT  ARLTPLEACA  HSFFDELRDP  NV-------K  LPNG--RDTP  ALFNFTTQEL  367
```

- Ser21 for *Hs*GSK3α and Ser9 for *Hs*GSK3β
- Tyr216 in *Hs*GSK3β or Tyr211 in *Tg*GSK3 activation loop
- Cys213 in *Tg*GSK3 activation loop
- Ser257 in *Tg*GSK3
- Gatekeeper residue
- DFG motif
- Activation loop

Supplementary Figure 2. Effect of LY2090314 treatment on wild-type and resistant mutants of *T. gondii* tachyzoites, and GSK3 protein sequence alignment. (a) Fluorescence microscopy showing intracellular growth of *T. gondii* LY2090314-resistant lines. HFF cells were infected by the indicated *T. gondii* strains in the presence or absence of 600 nM LY2090314. At 24 h post-infection, cells were fixed and stained with antibodies against GAP45 (magenta) and Hoechst (blue) to detect the inner membrane complex (IMC) of parasites and nuclei, respectively. (b) Multiple sequence alignment of GSK3 kinases from *T. gondii* (*Tg*), *C. parvum* (*Cp*), *P. falciparum* (*Pf*), and *H. sapiens* (*Hs*) highlights conserved features and unique residues. The phosphorylated serine residues of *Hs*GSK3α and *Hs*GSK3β are marked with grey dots. Conserved and phosphorylated tyrosine residues in the GSK3 activation loop are indicated with green dots. A conserved cysteine residue, which forms a disulfide bond in the *Tg*GSK3 crystal structure, is represented by a yellow dot. An Apicomplexa-specific serine residue at position 257 is highlighted with a green square. The main regions of the kinases are color-coded: Gatekeeper residue (magenta), DFG motif (blue), and activation loop (orange).

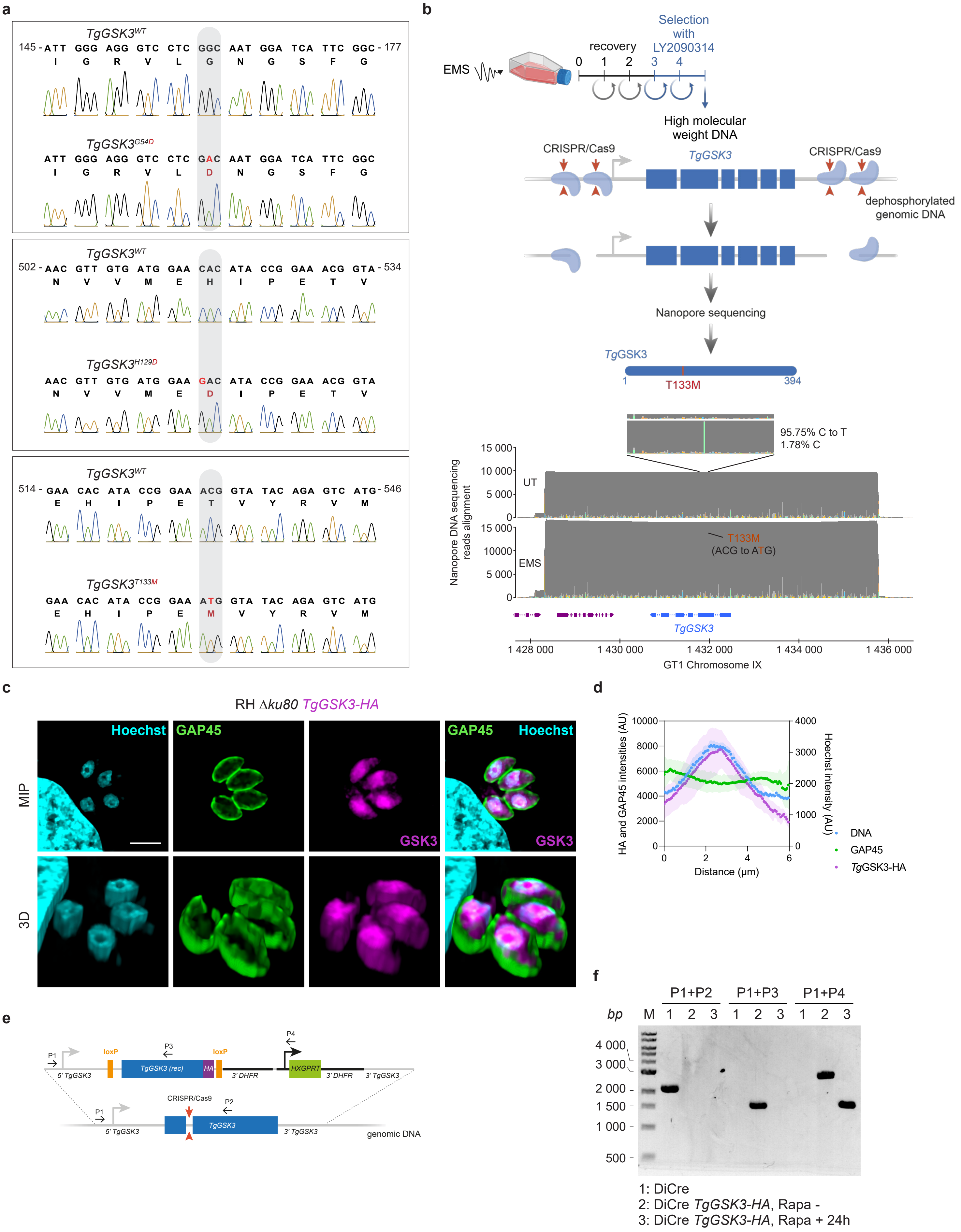
a
TgGSK3WT
145 - ATT GGG AGG GTC CTC GGC AAT GGA TCA TTC GGC - 177
I G R V L G N G S F G
TgGSK3G54D
ATT GGG AGG GTC CTC GAC AAT GGA TCA TTC GGC
I G R V L D N G S F G
TgGSK3WT
502 - AAC GTT GTG ATG GAA CAC ATA CCG GAA ACG GTA - 534
N V V M E H I P E T V
TgGSK3H129D
AAC GTT GTG ATG GAA GAC ATA CCG GAA ACG GTA
N V V M E D I P E T V
TgGSK3WT
514 - GAA CAC ATA CCG GAA ACG GTA TAC AGA GTC ATG - 546
E H I P E T V Y R V M
TgGSK3T133M
GAA CAC ATA CCG GAA ATG GTA TAC AGA GTC ATG
E H I P E M V Y R V M
b
EMS
recovery
Selection with LY2090314
0 1 2 3 4
High molecular weight DNA
CRISPR/Cas9
TgGSK3
CRISPR/Cas9
dephosphorylated genomic DNA
Nanopore sequencing
TgGSK3
1
T133M
394
95.75% C to T
1.78% C
Nanopore DNA sequencing reads alignment
15 000
10 000
5 000
0
UT
EMS
T133M
(ACG to ATG)
TgGSK3
1 428 000
1 430 000
1 432 000
1 434 000
1 436 000
GT1 Chromosome IX
c
RH Δku80 TgGSK3-HA
Hoechst
GAP45
GSK3
GAP45 Hoechst
MIP
3D
d
HA and GAP45 intensities (AU)
Hoechst intensity (AU)
Distance (µm)
DNA
GAP45
TgGSK3-HA
e
P1
loxP
P3
TgGSK3 (rec)
HA
loxP
3' DHFR
P4
HXGPRT
3' DHFR
3' TgGSK3
5' TgGSK3
CRISPR/Cas9
P2
TgGSK3
3' TgGSK3
genomic DNA
f
P1+P2
P1+P3
P1+P4
bp
M 1 2 3 1 2 3 1 2 3
4 000
3 000
2 000
1 500
1 000
500
1: DiCre
2: DiCre TgGSK3-HA, Rapa -
3: DiCre TgGSK3-HA, Rapa + 24h

Supplementary Figure 3. Analysis of LY2090314-resistant mutants, Subcellular localization of *Tg*GSK3, and Conditional knockout strategy. (a) Sanger sequencing validation of *TgGSK3* editing. Chromatograms of *TgGSK3* DNA sequences from parental (WT) and engineered parasites ($TgGSK3^{G54D}$, $TgGSK3^{H129D}$, and $TgGSK3^{T133M}$) are shown. Nucleotide positions relative to the ATG start codon on genomic DNA are indicated. (b) Workflow and analysis of *T. gondii* EMS mutagenesis combined with Cas9-mediated Nanopore sequencing of the *TgGSK3* genomic region. The lower panel displays a multi-way pileup alignment of Nanopore sequencing reads obtained from DNA extracted from untreated parasites (UT) and parasites treated with EMS and subsequently selected with LY2090314. A close-up view of the Nanopore DNA sequencing data is presented above the pileup, highlighting the ACG-to-ATG substitution (T133M mutation) identified exclusively in the EMS-treated, LY2090314-selected sample. Positive-strand genes are shown in magenta, and negative-strand genes are displayed in blue. Created in BioRender. Hakimi, M. (2025) https://BioRender.com/j19f292. (c) Fluorescence microscopy analysis of the RH *Tg*GSK3-HF strain. Tachyzoites were fixed 18 hours post-infection and stained with anti-GAP45 antibodies (magenta) and anti-HA antibodies (green). Images represent maximum projections of deconvolved z-stacks, with the scale bar indicating 5 µm. The lower panel displays side views of the 3D image reconstructions. (d) Quantification of fluorescence intensity signals of intracellular RH *Tg*GSK3-HF tachyzoites, obtained from immunofluorescence microscopy images. Arbitrary fluorescence units (AU) for HA-tagged *Tg*GSK3 (green), GAP45 (magenta), and Hoechst-stained DNA (blue) are shown. Data are presented as means ± SD represented as shaded error envelopes from n = 10 tachyzoites. (e) Schematic representation of the DiCre/loxP-based inducible knock-out strategy used for the conditional depletion of *Tg*GSK3, as depicted in Figure 3g. The positions of primers used for analytical PCR are indicated. (f) PCR analysis (P1+P2 and P1+P3) demonstrate correct allelic replacement at the endogenous *TgGSK3* locus in recombinant parasites (RH DiCre *Tg*GSK3) with the *Tg*GSK3 gene flanked by loxP sites (orange). PCR products from primer pairs P1+P3 and P1+P4 confirm excision of the floxed *TgGSK3* sequence.

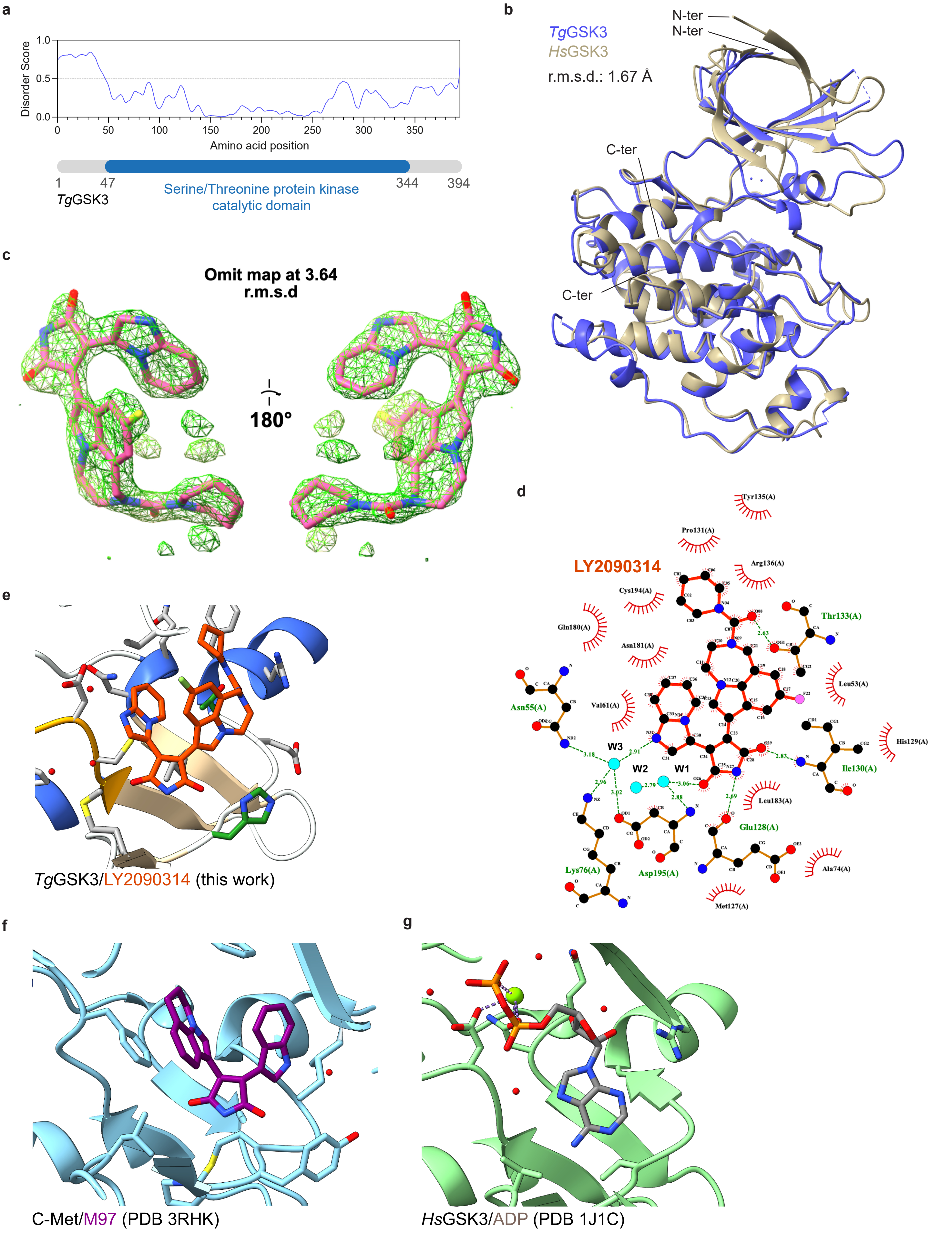

a
1.0
0.5
0.0
Disorder Score
0
50
100
150
200
250
300
350
Amino acid position
1
47
344
394
TgGSK3
Serine/Threonine protein kinase catalytic domain
b
TgGSK3
HsGSK3
r.m.s.d.: 1.67 Å
N-ter
N-ter
C-ter
C-ter
c
Omit map at 3.64 r.m.s.d
180°
d
LY2090314
Tyr135(A)
Pro131(A)
Arg136(A)
Cys194(A)
Thr133(A)
Gln180(A)
Asn181(A)
Leu53(A)
Asn55(A)
Val61(A)
His129(A)
W3
W2
W1
Ile130(A)
Leu183(A)
Glu128(A)
Lys76(A)
Asp195(A)
Ala74(A)
Met127(A)
3.18
2.91
2.96
3.02
2.79
3.06
2.88
2.69
2.83
2.63
e
TgGSK3/LY2090314 (this work)
f
C-Met/M97 (PDB 3RHK)
g
HsGSK3/ADP (PDB 1J1C)

Supplementary Figure 4. *Tg*GSK3 conservation and LY2090314 binding mode. (a) Schematic representation of *Tg*GSK3 protein architecture. Disorder scores, ranging from 0 (fully ordered) to 1 (fully disordered), were obtained using IUPred3. (b) Cartoon representations of the secondary structures of *Tg*GSK3 (blue; Protein Data Bank [PDB] ID: 9HVX) and *Hs*GSK3 (grey; PDB ID: 7B6F) are superposed, highlighting their high structural similarity. The overall root-mean-square deviation (r.m.s.d.) between the two structures is 1.67 Å, indicating conserved architectural features. (c) Omit map of LY2090314, calculated in Phenix and displayed as a green mesh over the stick representation of LY2090314. (d) Ligplot 2d interaction network of *Tg*GSK3 with LY2090314. LY2090314 is shown in red while water molecules are shown in cyan. (e/f/g) Split view of superposed crystal structures of *Tg*GSK3 bound to LY2090314 (e), *Hs*C-Met kinase bound to M97 (f) and *Hs*GSK3 bound to ADP (g).

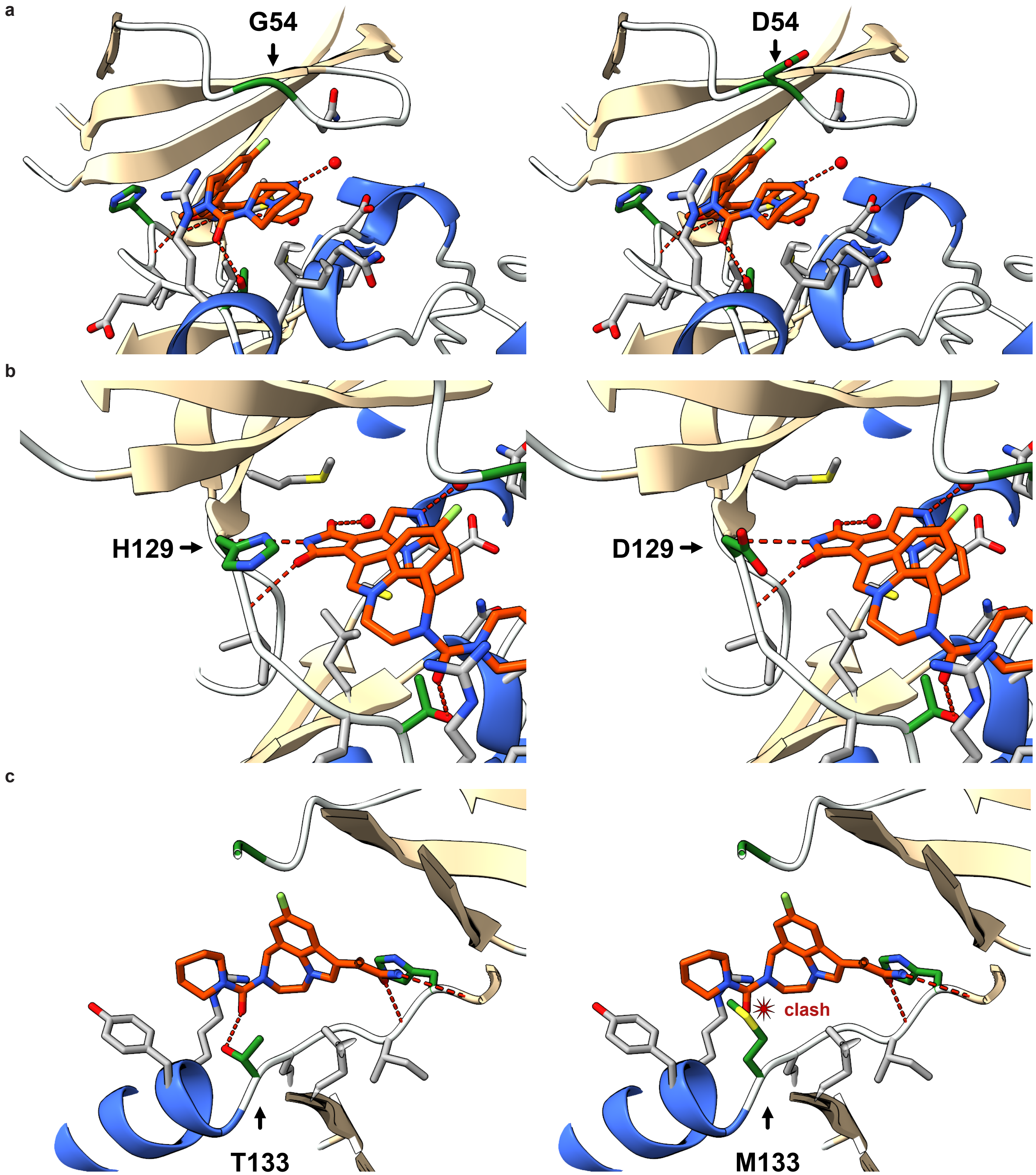
a
G54
D54
b
H129
D129
c
T133
M133
clash

Supplementary Figure 5. Predicted structural changes of the LY2090314 resistance conferring mutations. Original structural observations are shown on the left while mutation substitutions are on the right for G54D (a), H129D (b), and T133M (c) mutants. Color and feature representation rules are the same as those used in Figure 4b.

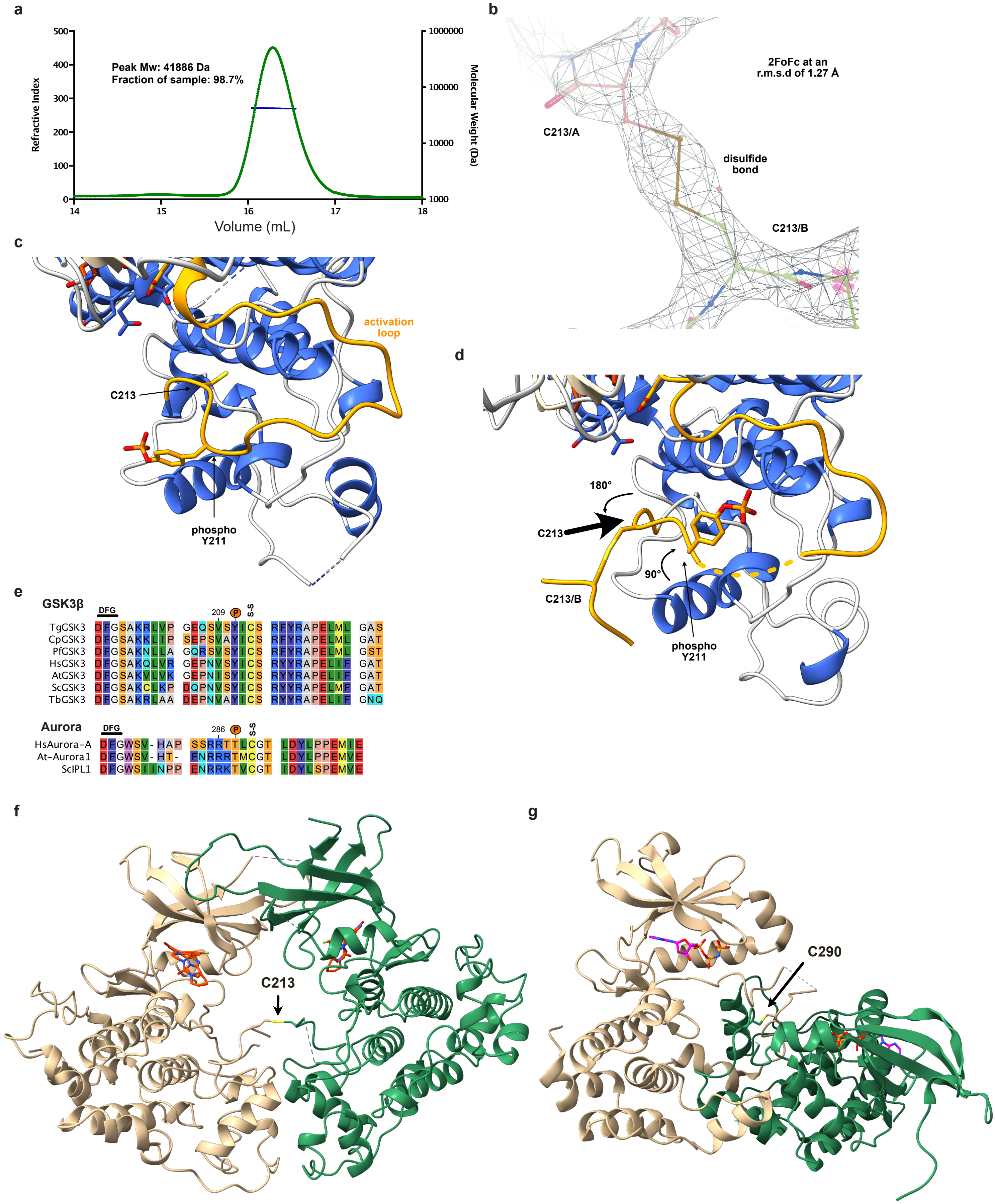

a
Peak Mw: 41886 Da
Fraction of sample: 98.7%
Refractive Index
Molecular Weight (Da)
Volume (mL)
b
2FoFc at an
r.m.s.d of 1.27 Å
C213/A
disulfide
bond
C213/B
c
activation
loop
C213
phospho
Y211
d
180°
C213
90°
C213/B
phospho
Y211
e
GSK3β
DFG
209
P
S-S
TgGSK3
CpGSK3
PfGSK3
HsGSK3
AtGSK3
ScGSK3
TbGSK3
Aurora
DFG
286
P
S-S
HsAurora-A
At-Aurora1
ScIPL1
f
C213
g
C290

Supplementary Figure 6. Crystal structure of cysteine adduct *Tg*GSK3 dimer. (a) Recombinant *Tg*GSK3 is found as a monomer in solution as determined by size exclusion chromatography coupled to laser light scattering (SEC-MALLS). OMNISEC chromatogram displaying the refractive index as a function of elution volume in green while the calculated mass within the peak region is displayed in blue. The run was performed with a S200 10/300 GL increase column. (b) 2FoFc electron density of the disulfide bridge at an r.m.s.d of 1.27 Å as depicted in coot. (c) Local schematic view of the activation loop within the monomeric structure of *Tg*GSK3. The activation loop is shown in yellow while the side chains of the phosphotyrosine 211 and cysteine 213 are depicted. (d) Local schematic view of the activation loop within the Cys213/ss/Cys213 dimeric structure of *Tg*GSK3. Using the same representation as in (c), special highlight is shown for the structural re-arrangements undergone to form the disulfide bridge. (e) Sequence conservation of the activation loop disulfide bond forming cysteines in GSK3β and Aurora. Above, the local sequence alignment of the activation loop N-terminus for GSK3β within *Toxoplasma gondii* (*Tg*), *Plasmodium falciparum* (*Pf*), *Cryptosporidium parvum* (*Pv*), *Homo sapiens* (*Hs*), *Arabidopsis thaliana* (*At*), *Saccharomyces cerevisiae* (*Sc*) and *Trypanosoma brucei* (*Tb*). Below, the same region is shown for Aurora within *Homo sapiens*, *Arabidopsis thaliana* and *Saccharomyces cerevisiae*. In both cases, the DFG motif, the phosphorylated tyrosine or serine and disulfide bonding cysteine are schematically highlighted. (f) Disulfide linked dimer of *Tg*GSK3 with the monomer A in tan and monomer B in sea green. LY2090314 is also depicted in an orange stick fashion while the disulfide bridge is shown with an arrow. (g) Disulfide linked Aurora dimer (PDB ID: 6VPJ) using the same color code as in F while structurally aligning the monomer A to *Tg*GSK3 dimer to highlight the rotation of monomer B.
