# Supplementary material for: Structural and functional characterization of TgGSK3, a druggable kinase in Toxoplasma gondii
Source: Nat Commun. 2025 Nov 5;16:9765. doi: 10.1038/s41467-025-64701-7 (PMC12589562; doi:10.1038/s41467-025-64701-7)
Supplement: Supplementary file 2 — Description Of Additional Supplementary File [file 41467_2025_64701_MOESM2_ESM.pdf]

Description of Additional supplementary files

Supplementary Data 1

Strains and plasmids; Primers, oligonucleotides, and crRNA

Supplementary Data 2

Transcriptomic analysis and variant calling of LY2090314-resistant mutants and the parental 8 strain by Illumina sequencing, and Cas9-targeted sequencing by Nanopore.

Supplementary Data 3

MS-based proteomic characterization of TgGSK3 proxisome.

Supplementary Data 4

TgGSK3 interactome identified by yeast two-hybrid (Y2H) screening using a T. gondii cDNA library as prey and the catalytically inactive N-terminal TgGSK3 13 K76H mutant as bait.

Supplementary Data 5

AlphaFold-Multimer Analysis of interactors identified by Yeast Two-Hybrid and proximity 16 labeling of TgGSK3, and Analysis of AlphaFold-Multimer Predictions.
